# Spectral Computed Tomography-Derived Iodine Content and Tumor Response in the Follow-Up of Neuroendocrine Tumors—A Single-Center Experience

**DOI:** 10.3390/curroncol30020115

**Published:** 2023-01-23

**Authors:** Winna Lim, Elisa Birgit Sodemann, Laura Büttner, Martin Jonczyk, Willie Magnus Lüdemann, Johannes Kahn, Dominik Geisel, Henning Jann, Annette Aigner, Georg Böning

**Affiliations:** 1 Department of Radiology, Charité—Universitätsmedizin Berlin, Corporate Member of Freie Universität Berlin, Humboldt-Universität zu Berlin, and Berlin Institute of Health, Augustenburger Platz 1, 13353 Berlin, Germany; 2 Institute of Neuroradiology, Charité—Universitätsmedizin Berlin, Corporate Member of Freie Universität Berlin, Humboldt-Universität zu Berlin, and Berlin Institute of Health, Augustenburger Platz 1, 13353 Berlin, Germany; 3 Department of Hepatology and Gastroenterology, Charité—Universitätsmedizin Berlin, Corporate Member of Freie Universität Berlin, Humboldt-Universität zu Berlin, and Berlin Institute of Health, Augustenburger Platz 1, 13353 Berlin, Germany; 4 Institute of Biometry and Clinical Epidemiology, Charité—Universitätsmedizin Berlin, Corporate Member of Freie Universität Berlin, Humboldt-Universität zu Berlin, and Berlin Institute of Health, Campus Charité Mitte, Charitéplatz 1, 10117 Berlin, Germany

**Keywords:** spectral computed tomography, neuroendocrine tumor, computed tomography, NET

## Abstract

Spectral computed tomography (SCT) allows iodine content (IC) calculation for characterization of hypervascularized neoplasms and thus might help in the staging of neuroendocrine tumors (NETs). This single-center prospective study analyzed the association between SCT-derived IC and tumor response in the follow-up of metastasized NETs. Twenty-six patients with a median age of 70 years (range 51–85) with histologically proven NETs and a total of 78 lesions underwent SCT for staging. Because NETS are rare, no primary NET types were excluded. Lesions and intralesional hotspots were measured in virtual images and iodine maps. Tumor response was classified as progressive or nonprogressive at study endpoint. Generalized estimating equations served to estimate associations between IC and tumor response, additionally stratified by lesion location. Most commonly affected sites were the lymph nodes, liver, pancreas, and bones. Median time between SCT and endpoint was 64 weeks (range 5–260). Despite statistical imprecision in the estimate, patients with higher IC in lymphonodular metastases had lower odds for disease progression (adjusted OR = 0.21, 95% CI: 0.02–2.02). Opposite tendencies were observed in hepatic and pancreatic metastases in unadjusted analyses, which vanished after adjusting for therapy and primary tumor grade.

## 1. Introduction

Contrast-enhanced computed tomography (CT) is a standard and readily available imaging modality for the follow-up of patients with neuroendocrine tumors (NETs). As CT technology advances, spectral CT (SCT) is becoming a promising option in oncologic imaging. It has already been shown that signal-to-noise ratio und contrast-to-noise ratio increase in low-energy virtual monochromatic images, thus improving diagnostic confidence [1]. SCT also allows differentiation of materials in imaging. Therefore, iodine content (IC) derived from SCT may be particularly useful in oncologic imaging, specifically in the characterization of hypervascularized neoplasms.

Most NETs have abundant vasculature, seen as hypervascularization in imaging [2]. NETs are rare and have a wide range of clinical manifestations. While they mostly occur in the gastrointestinal tract and pancreas, NETs only account for up to 1.5% of gastroenteropancreatic neoplasms [3]. Clinical imaging plays a major role in the therapeutic management of patients with NETs, both for baseline workup prior to therapy and for follow-up after initial treatment. The main goals of follow-up imaging are to detect disease progression and/or to assess treatment response [2]. This is important to ensure optimal therapeutic management, particularly in patients with progressing low- and intermediate-grade NETs. These groups of NETs tend to grow slowly, and disease progression might therefore be missed.

This study aimed to explore the usability of SCT parameters in the follow-up of patients with NETs focusing on the association to tumor response.

## 2. Materials and Methods

### 2.1. Study Population and Study Design

**Figure 1:**
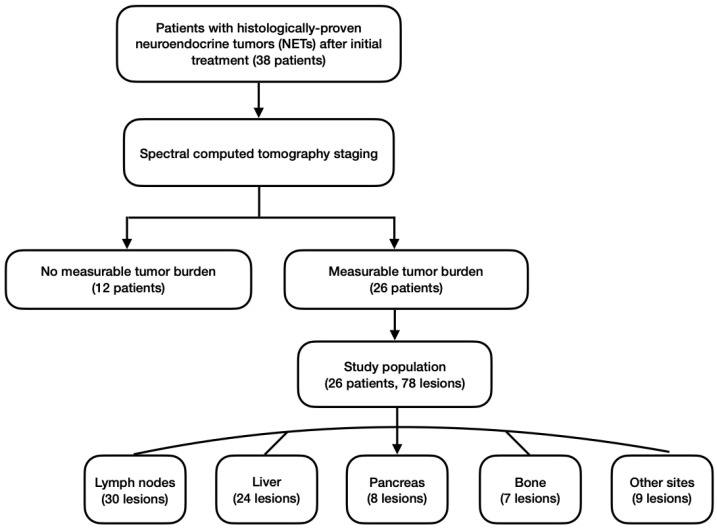
Flowchart of study population and lesions analyzed.

Patients with histologically proven NETs who either had a change in therapeutic management beforehand (e.g., after surgery or because of disease progression) or were managed by watch–and-wait without medication were prospectively scheduled for follow-up staging using SCT, if magnetic resonance imaging (MRI) was not possible for some reason (claustrophobia, non-MRI-safe cardiac devices, etc.). Follow-up was performed until a change in therapy for any reason occurred. Possible reasons included disease progression, surgery, therapy intolerance, or complications. Otherwise, the last follow-up available for a patient in the database was taken as study endpoint. The inclusion criterion was any type of NET with at least one measurable tumor manifestation in the SCT, including lymphonodular and/or distant metastases. The final study population of this single-center study consisted of 26 patients with histologically proven NETs and a total of 78 lesions who underwent SCT for staging from February 2017 to August 2021 (Figure 1).

### 2.2. Scan Protocol

SCT was performed in a second-generation spectral multislice CT scanner (Revolution HD, GE Healthcare, Milwaukee, WI, USA) using Gemstone Spectral Imaging (GSI) with ultra-fast 80/140 kV switching. Detailed scan parameters are listed in Table 1. After body-weight-adapted intravenous injection of non-ionic contrast agent, only the arterial phase was scanned with the GSI technique. Scans were acquired 18 s after contrast agent injection for late arterial phase, 53 s for portal venous phase, if performed, and 133 s for venous phase acquisitions [4].

### 2.3. Image Analysis

**Figure 2:**
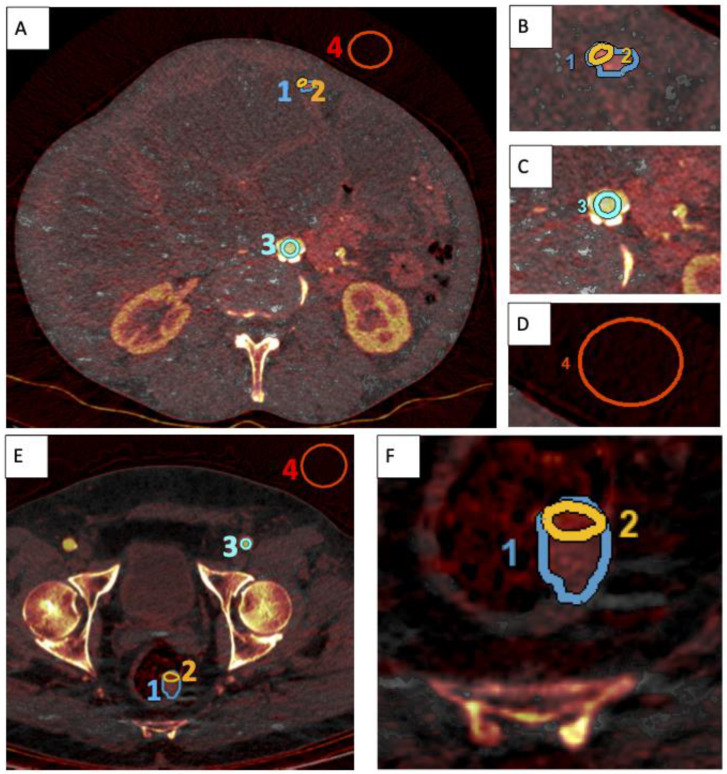
Placement of ROIs. (**A**) is an SCT iodine map to illustrate ROI placement for analysis of a peritoneal implant in a 64-year-old man with peritoneal implants following surgical removal of a pancreatic NET. (**B**–**D**) are magnified details of the ROIs placed in (**A**). (**E**) illustrates ROI placement in the analysis of a rectal lesion in an SCT iodine map of a 65-year-old man. (**F**) is a magnified detail of (**E**) to highlight placement of the tumor lesion ROI and hotspot ROI in this case. ROIs are labeled as follows, blue (1): total-lesion ROI; orange (2): intralesional-hotspot ROI; turquoise (3): arterial ROI; red (4): air ROI.

GSI Volume Viewer (GE Healthcare) was used to generate virtual monochromatic images (VMI) between 40 and 140 keV. An Advantage Workstation (GE Healthcare) was used to analyze lesion parameters. Regions of interest (ROIs) were manually placed in the clinical standard 70 keV equivalent image: one ROI encompassing each tumor lesion, another around intralesional ‘hotspots’, an arterial ROI (e.g., aorta, common iliac artery or common femoral artery), and a ROI in surrounding air. These ROIs were semiautomatically copied into the 40, 70, and 140 keV images as well as into iodine maps (Figure 2). Iodine concentration (IC) was determined from the total lesion ROI and the hotspot ROI. Normalized iodine content (NIC) was calculated for lesions and hotspots by dividing each IC and hotspot IC by the IC in the arterial ROI [4].

$$\mathrm{Normalized}\;\mathrm{iodine}\;\mathrm{content}\;\mathrm{of}\;\mathrm{lesion}\;=\frac{{\;\mathrm{Iodine}\;\mathrm{content}\;}_{\mathrm{lesion}}}{{\;\mathrm{Iodine}\;\mathrm{content}\;}_{\mathrm{artery}}}$$

$$\mathrm{Normalized}\;\mathrm{iodine}\;\mathrm{content}\;\mathrm{of}\;\mathrm{hotspot}=\frac{{\;\mathrm{Iodine}\;\mathrm{content}\;}_{\mathrm{hotspot}}}{{\mathrm{Iodine}\;\mathrm{content}\;}_{\mathrm{artery}}}$$

Attenuation slope was defined as the difference in mean attenuation between 40 keV and 140 keV divided by 100 [4]. All clinical and radiological follow-up (CT or MRI) data obtained from the study patients during the interval from SCT to the endpoint were analyzed. We used the Response Evaluation Criteria in Solid Tumors (RECIST) 1.1 [5] but applied them on a per-lesion level and categorized each lesion at study endpoint as progressive disease (PD) versus nonprogressive disease (combining stable disease (SD) and partial response (PR)). Complete response did not occur in our study population.

### 2.4. Statistical Analysis and Graphical Abstract

To describe the dataset, categorical variables are presented as absolute frequencies and percentages, continuous data as median and interquartile range (IQR) or with a range, where appropriate. Normalized iodine content is graphically displayed to assess its associations with tumor grade and tumor response. Generalized estimating equations for logistic regression were used to assess the association between IC and tumor response (PD vs. SD/PR) for each lesion, to account for the dependency of lesions clustered within one patient, and to account for differences between lesion locations. These models were adjusted for treatment and primary tumor grade, and additionally were run stratified by lesion location for those locations with a sufficient number of observations and events. All statistical analyses were conducted with R [6] using RStudio (Version 2022.7.1.554) [7] and relevant R packages [8,9,10,11,12,13]. Graphical abstract was created with BioRender.com.

## 3. Results

### 3.1. Characteristics of Study Population

Twenty-six patients (12 male, 14 female; median age 70 years, range 51–85 years) with a total of 78 lesions were included in the study (Table 2). Forty-three lesions (55%) were located in the small intestine as the primary tumor site, followed by the pancreas (20 lesions, 26%). The median interval between SCT and last follow-up was 64 weeks (range 5–260). Lesions had a median area of 212 mm^2^ (IQR 72; 382).

At the end of the study, 30 lesions (38%) were classified as progressive and 48 lesions as nonprogressive: 36 lesions (46%) were stable, and 12 lesions (15%) were found to show a partial response (Table 3). Median NIC was higher in progressive than in nonprogressive lesions. There was no relevant difference in the attenuation slope between both groups. Metastases were mostly found in lymph nodes (30 lesions, 38.5%), liver (24 lesions, 30.8%), pancreas (8 lesions, 10.3%), and bones (7 lesions, 9%). Thirty-two patients had low-grade (G1), 35 patients had intermediate-grade (G2), and 11 patients had high-grade (G3) primary NETs.

Therapeutic decisions were made by an interdisciplinary tumor board, taking all available patient information into account. The therapeutic strategies included watch-and-wait without medication (43.59%, *n* = 34 lesions), somatostatin analogues (30.77%, *n* = 24 lesions), mTOR inhibitor (everolimus) (7.69%, *n* = 6 lesions), chemotherapy (11.54%, *n* = 9 lesions), and a combination of different chemotherapies (6.41%, *n* = 5 lesions). Only pancreatic NET patients were treated with chemotherapy. Intestinal NETs were most commonly treated with somatostatin analogue (SSA) (Table 4).

NIC was higher in lesions under watch and wait (without medication). Almost all lesions originating from high-grade primary tumor were under treatment. Lesions with no therapy originated mostly from intestinal NET (Table 5). 

### 3.2. Spectral CT Parameters

**Figure 3:**
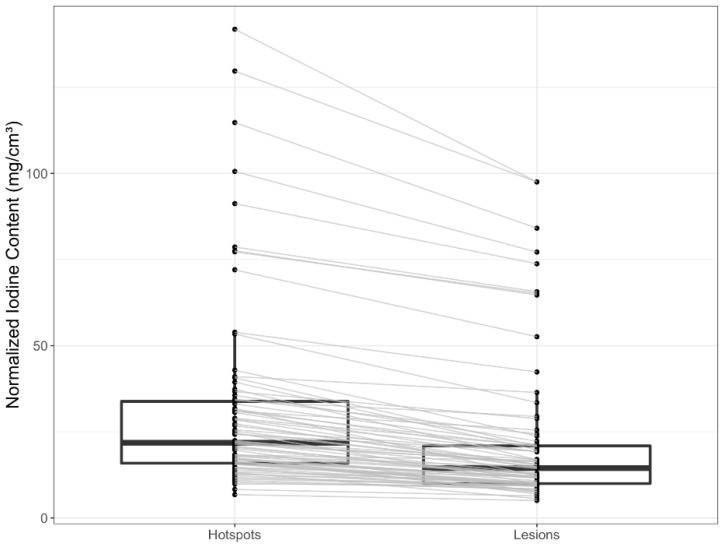
Boxplots of normalized iodine content across intralesional hotspots and total lesions. Each lesion is connected to its intralesional hotspot with a gray line.

**Figure 4:**
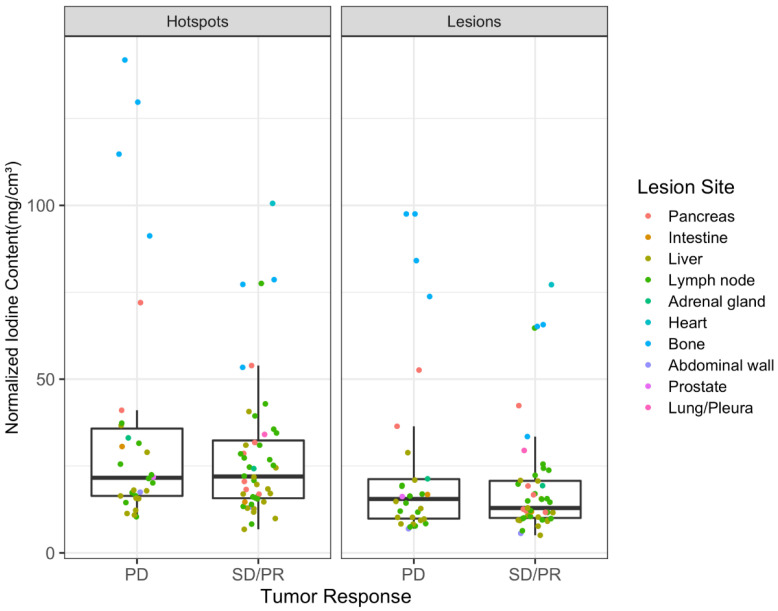
Boxplots of normalized iodine content across progressive (PD) and nonprogressive lesions (SD/PR). Individual data points are depicted as dots. Dots are colored based on lesion localization in the body. PD: progressive disease, SD: stable disease, PR: partial response.

**Figure 5:**
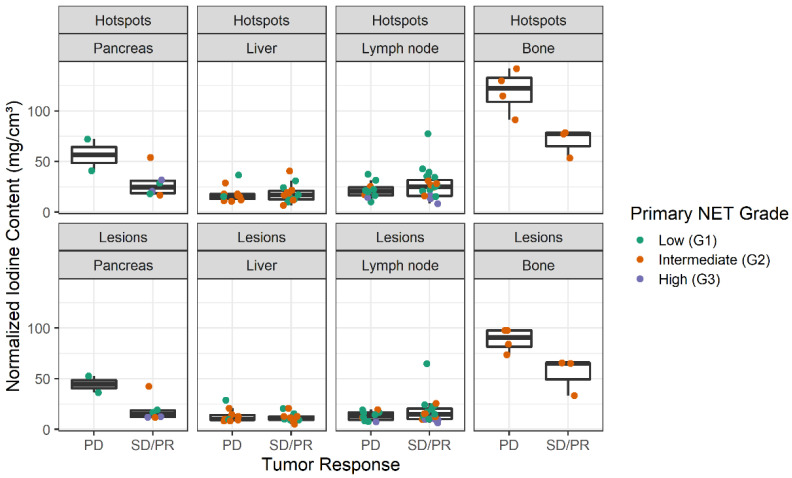
Boxplots of normalized iodine content across lesions and hotspots in four of the most prevalent lesion sites (pancreas, liver, lymph nodes, and bone). Individual data points are depicted as dots. Dots are colored based on primary tumor grades. PD: progressive disease, SD: stable disease, PR: partial response.

Intralesional hotspots had higher IC than the total lesions (Figure 3). However, comparing IC between the two groups of tumor response status, there was visually no relevant difference in central tendencies between PD and SD/PR in hotspots (Figure 4). Bone lesions had the highest NIC, followed by pancreatic lesions (Figure 4). Regardless of their tumor response status, all bone lesions had higher NIC compared to lesions in other sites. Nonprogressive pancreatic lesions had NIC similar to other lesions (Figure 5). Distribution of NIC in each lesion across the total patient population is found in Figure S1.

The largest differences in NIC between PD vs. SD/PR were found for pancreatic and bone lesions, with progressive lesions having clearly higher NIC than nonprogressive lesions (Figure 5). In the subsets of liver and lymph node lesions, there were no relevant differences in NIC between progressive and nonprogressive lesions. Conversely, progressive lymph node lesions had slightly lower NIC than nonprogressive lymph node lesions. High-grade (G3) primary tumors were only present in the pancreas and lymph nodes, and generally had lower NIC than low- and intermediate-grade primary tumor lesions.

**Figure 6:**
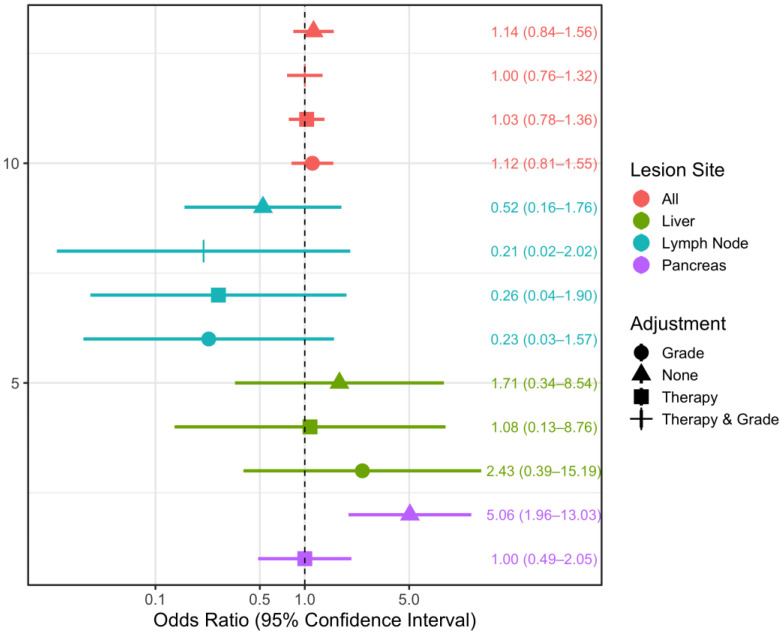
Odds ratio estimates for the association between normalized iodine content and tumor response, based on generalized estimating equations logistic regression, taking clustering within patients into account. Lesions were stratified by lesion location and different adjustment sets, wherever possible due to limitations in sample size. OR: odds ratio, CI: confidence interval.

**Figure 7:**
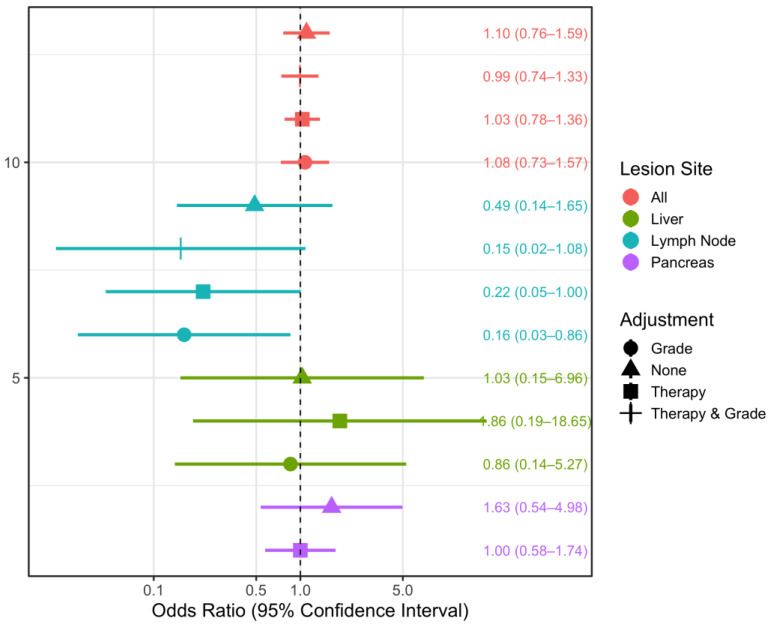
Odds ratio estimates for the association between attenuation slope and tumor response, based on generalized estimating equations logistic regression, taking clustering within patients into account. Lesions were stratified by lesion location and different adjustment sets, wherever possible due to limitations in sample size. OR: odds ratio, CI: confidence interval.

Using generalized estimating equations for logistic regression, we found that adjustment for therapy (any therapy vs. no therapy/watch and wait) accounted for the association between NIC and progressive disease in the total study population, as well as in the subsets of liver and pancreatic lesions (Figure 6). In the total population, where localization and patient-clustered lesions were considered, an increase in NIC of 10 mg/cm^3^ was associated with a 1.14-fold higher risk of progressive disease (OR = 1.14 (95% CI: 0.84–1.56)) when no adjustment was made. When therapy as well as primary tumor grade were adjusted for, no discernible association between NIC and outcome (PD) was observed (adjusted OR = 1.00 (95%CI 0.76–1.32)) (Figure 6). Conversely, in the subset of lymph node lesions, we found protective effects of a higher NIC, independent of adjustment. In this subset, when adjusted for primary tumor grade and therapy, an increase in NIC by 10 mg/cm^3^ was associated with a 79% lower risk of progressive disease (adjusted OR = 0.21 (95%CI 0.02–2.02)). The same statistical approach was used to assess possible associations between attenuation slopes and PD (Figure 7). Especially for hepatic and pancreatic lesions, estimated associations were rather weak. The results for NIC and attenuation slopes were found to be highly similar for the total population and the subset of lymph node lesions. A difference was only found in the subset of liver lesions when adjustment was made for primary tumor grade, which revealed an increase in NIC to be a risk factor for PD (OR = 2.43 (95%CI 0.39–15.19)), while an increase in the attenuation slope was found to be protective (OR = 0.86 (95%CI 0.14–5.27)). However, due to the small sample size, the precision of all estimates is low, and generalizations cannot be derived.

## 4. Discussion

NET hypervascularization has been shown to characterize both primary tumors and their abdominal metastases, including hepatic and lymph node metastases [14]. This hypervascularization can be detected by CT, MRI, and contrast-enhanced ultrasound [14]. MRI is superior in imaging hepatic and other parenchymal metastases, which was also reflected in some of the patients in our study population who underwent MRI staging to better visualize hepatic metastases. However, CT is more widely available, less expensive, and is superior for simultaneous thoracic and abdominal staging. In a study conducted in 2021, the authors [4] did not find associations between tumor response and SCT markers in neuroendocrine neoplasms, attributing this result to the diversity of therapeutic regimens before SCT in their study population as well as changes in therapy during the follow-up period in about 23% of cases. Therapeutic management in the study ranged from various systemic therapies to surgery and radiological interventions [4]. In the present study, we therefore addressed this issue by creating a study population with more homogeneous therapeutic management.

SCT is unique in allowing quantitative evaluation of iodine content in the tumor substrate. Iodine content depends on tumor vascularization. However, the characteristic hypervascularization of NETs can be affected by several factors such as treatment, tumor grade and presumably the localization of both primary and metastatic lesions.

### 4.1. Therapy

The ability of SCT to predict tumor response is typically taken advantage of for baseline imaging before treatment. In this setting, SCT was found to have a predictive potential in hypopharyngeal and laryngeal neoplasms [15]. In 2019, a study showed possible predictive potential in non-small-cell lung cancer [16]. Both studies investigated pretherapeutic SCT in patients with advanced and non-resectable neoplasms subsequently treated by chemoradiotherapy. However, the predictive potential in the follow-up of tumors after primary treatment is just as important to detect early progression and ensure timely treatment. In the study presented here, we therefore investigated patients with histologically-proven NETs after initial treatment. Treatments in our study population included chemotherapy, SSA, mTOR inhibitor, and combinations of chemotherapy, SSA, and telotristat ethyl (Xermelo^®^, TerSera Therapeutics LLC, Deerfield, IL, USA); combination treatment is prescribed to provide antiproliferative (chemotherapy) and antisecretory effects (SSA and Xermelo^®^). As shown in Table 5, median NIC is higher in lesions without therapy (watch and wait) than in lesions with any kind of therapy. Thirty-four lesions in our study were in patients without medical therapy, and 68% (*n* = 23) of them were classified as a PD at study endpoint (Table 3). Thus, treatment of primary tumors and the medications patients were taking during the study period might have modified tumor angiogenesis and consequently its vascularization. This in turn might have affected the potential of SCT to predict therapeutic responses. It would be interesting to explore the usefulness of SCT prior to treatment in advanced NETs or in patients with unresectable advanced NET metastases, similar to the above-mentioned studies—ideally comparing SCT with MRI, since MRI has also been reported to have predictive potential in NETs [17,18].

### 4.2. Tumor Grade

Pancreatic and lymph node lesions originating from high-grade NET primaries tended to have lower IC compared to lesions from low- and intermediate-grade primaries arising in the same organs (Figure 5). This could be related to the fact that high-grade tumors (G3) contain less well-differentiated cells than low- and intermediate-grade tumors. Poor cell differentiation affects the formation of adequate new vessels. Consequently, poor development of vascular structures leads to lower iodine uptake. This finding also confirmed the results of previous studies, which found hypovascularization in up to 42% of intermediate- and high-grade pancreatic NETs [17,19,20]. For example, in 2021, Li et al. investigated the potential of SCT for differentiating low-grade (G1) from non-low-grade (G2 and G3) pancreatic NETs and found a lower iodine concentration in Grade 2 and 3 tumors when compared to Grade 1 tumors [21].

In our study, 40% of lesions that originated from low-or intermediate-grade NETs but only 10% of lesions originating from high-grade NETs were classified as progressive at the study endpoint. This might be attributable to the fact that almost all patients with high-grade lesions had therapy of some kind (Table 5), while roughly half of the low- and intermediate-grade lesions were without medical treatment (watch and wait).

### 4.3. Lesion Sites

Arising from neuroendocrine cells, NETs can occur in every organ [2]. They are most common in the pancreas and small intestine [3], which is also reflected in the current study: 61% of the lesions spread from intestinal NETs and 29% from pancreatic NETs. Metastasis is a highly complex topic in terms of cell biology, with different organs providing different environmental conditions for metastatic cells [22]. In our study, NET metastases were mainly found in the following locations: lymph nodes (30 lesions, 38.5%), liver (24 lesions, 30.8%), pancreas (8 lesions, 10.3%), and bones (7 lesions, 9%). Much less common were metastases in other organs (*n* ≤ 2). Since the location of metastatic lesions could also have an impact on their vascularization, we also analyzed our results by organ site. This subanalysis revealed higher lymphonodular IC to be associated with a lower risk of disease progression, albeit with very low precision. This observation might be attributable to the fact that normal lymph nodes usually have hilar vascularity, while malignant lymph nodes can have peripheral or mixed vascularity [23]. Additionally, lack of iodine uptake might also be related to central necrotic changes of metastatic lymph nodes. Only the subsets of pancreatic and bone lesions were found to differ in NIC across tumor response groups in our analysis. However, there were only a few lesions in these subsets (*n* < 10 each). Only the pancreatic subset was large enough to be stratified in the generalized estimating equation for logistic regression. In this subset, there was no association at all between NIC and PD when adjusted for therapy. However, our observations should be interpreted with caution due to the small sample size and generally wide confidence intervals.

An earlier study of our group showed a significantly higher IC and attenuation slopes in primary tumors than in metastatic lesions [4]. It would be interesting to investigate if there is a difference in NIC between primary tumors and metastatic lesions in a similar study setting with a controlled treatment window.

### 4.4. Spectral Imaging

Dual-energy CT offers many benefits in the clinical setting and is particularly helpful in oncologic imaging such as differentiation of benign from malignant hepatic lesions [24] or improved diagnostic confidence in hypervascularized abdominal tumors [1,25,26]. Apart from dual-energy CT, the emerging technique of spectral photon-counting detector CT also allows material differentiation based on their specific energy signatures and can provide even more detailed and accurate information on the materials present in the scan through optimized spectral imaging [27]. Many studies have reported the potential of photon-counting detector CT in various clinical settings, including assessment of coronary artery plaque [28] or assessment of very small structures such as the ossicular chain [29]. In addition, virtual monochromatic images and iodine maps are superior for evaluating soft tissue structures, in both oncologic and infectiologic patients. For example, low-keV virtual monochromatic images and an iodine chart achieved higher diagnostic accuracy in differentiating between pleural effusion and pleural empyema [30] and low-keV virtual monochromatic images improved objective and subjective quality of scans in the arterial phase compared to conventional CT in oncologic imaging [31]. Since photon-counting detector CT has clear advantages over dual-energy CT, it would be interesting to investigate the diagnostic performance of photo-counting detector CT in a similar study design in the future.

### 4.5. Limitations

Despite our efforts to control therapeutic management in our study population, this is still an issue that shall be addressed in future study designs, for example by collecting more cases for each NET type and treatment combination in order to include enough lesions for statistical analyses. However, due to the rare occurrence of NETs and the heterogeneous nature of therapeutic approaches for these tumors, it is difficult to recruit enough patients with the same therapy regimens for meaningful subset analyses. Further limitations of this study include the heterogenous characteristics of the study population regarding primary tumor grade and lesion localization. The number of patients is small despite a >4-year recruitment period. Firstly, many patients scheduled for SCT had stable disease without any measurable tumor burden detectable in SCT scans. Secondly, some patients were scheduled for SCT but the examinations were not performed as planned (e.g., scanner defect, switch of staging modality to MRI).

## 5. Conclusions

In the follow-up of patients with initially treated metastasized neuroendocrine tumors, patients with higher IC in lymphonodular metastases had lower odds for disease progression (adjusted OR = 0.21, 95% CI: 0.02–2.02), despite statistical imprecision in the estimate. Opposite tendencies were observed in hepatic and pancreatic metastases in unadjusted analyses, which vanished after adjusting for therapy and primary tumor grade.

## Figures and Tables

**Table 1:** CT scan parameters.

Scan Phase	Arterial	Portal Venous and Venous	
Voltage	Dual-energy spectral mode (80/140 kVp)	Mono-energy mode (120 kVp)	
Postprocessing datasets	Iodine map and virtual monochromatic images (40 to 140 keV|10 keV increments)	Polychromatic images	
Adaptive statistical iterative reconstruction level	70%		
Noise index	21		
Pitch	1.375		
Collimation	64 × 0.625 mm		
Rotation time	0.7 s		
Tube current	Average 260–640 mA	Min/max: 100/500 mA	
Smart mA	On		
Auto mA	Off (not available from vendor)	On	
Reconstruction mode	Slice (axial)		
Reconstructed slice thickness	0.625 mm		
Reconstructed slice interval	0.625 mm		
FOV	Display FOV: patient-dependent Scanning FOV: 50 cm		

kVp: peak kilovoltage, keV: kiloelectronvolt, mm: millimeter, mA: milliampere, FOV: field of view.

**Table 2:** Patient characteristics.

Age	Min/Max	51.0/85.0
	Med [IQR]	70.0 [64.0; 74.0]
Gender	Female	14 (53.85%)
	Male	12 (46.15%)
	Total	26 (100.00%)
Location of NET primaries	Pancreas	20 (25.64%)
	Intestine	43 (55.13%)
	Prostate	1 (1.28%)
	Lung	6 (7.69%)
	Unclear primary	8 (10.26%)
	Total lesions	78 (100.00%)
Interval between SCT and study endpoint (weeks)	Min/Max	5.0/260.0
	Med [IQR]	64.0 [24.0; 103.0]

Min: minimum, Max: maximum, IQR: interquartile range, SCT: spectral computed tomography, SD: standard deviation.

**Table 3:** Lesion characteristics by disease status at the end of the study.

	Progressive Disease (*n* = 30)	Nonprogressive Disease (*n* = 48)	Total (*n* = 78)
Tumor response			
| Stable disease (0)	0 (0.0%)	36 (100.0%)	36 (100.0%)
| Progressive disease (1)	30 (100.0%)	0 (0.0%)	30 (100.0%)
| Partial response (2)	0 (0.0%)	12 (100.0%)	12 (100.0%)
Normalized iodine content (mg/cm^3^)			
| Median (IQR)	15.50 (9.86, 21.22)	12.92 (10.05, 20.73)	14.46 (9.99, 20.93)
Attenuation slope			
| Median (IQR)	1.45 (0.98, 2.20)	1.41 (1.04, 2.06)	1.41 (1.01, 2.09)
Therapy			
| Temozolomide and capecitabine (Tem./Cap.)	0 (0.0%)	6 (100.0%)	6 (100.0%)
| Everolimus	2 (33.3%)	4 (66.7%)	6 (100.0%)
| Somatostatin analogue (SSA)	5 (20.8%)	19 (79.2%)	24 (100.0%)
| Streptozocin and 5-fluorouracil	0 (0.0%)	3 (100.0%)	3 (100.0%)
| Tem./Cap., SSA, telotristat ethyl	0 (0.0%)	5 (100.0%)	5 (100.0%)
| Watch and wait (none)	23 (67.6%)	11 (32.4%)	34 (100.0%)
Therapy (binary)			
| Any	7 (15.9%)	37 (84.1%)	44 (100.0%)
| None (watch-and-wait)	23 (67.6%)	11 (32.4%)	34 (100.0%)
Primary tumor grade			
| Low-grade (G1)	12 (37.5%)	20 (62.5%)	32 (100.0%)
| Intermediate-grade (G2)	15 (42.9%)	20 (57.1%)	35 (100.0%)
| High-grade (G3)	3 (27.3%)	8 (72.7%)	11 (100.0%)
Lesion site			
| Pancreas	2 (25.0%)	6 (75.0%)	8 (100.0%)
| Intestine	1 (50.0%)	1 (50.0%)	2 (100.0%)
| Liver	10 (41.7%)	14 (58.3%)	24 (100.0%)
| Lymph node	10 (33.3%)	20 (66.7%)	30 (100.0%)
| Adrenal gland	1 (50.0%)	1 (50.0%)	2 (100.0%)
| Heart	0 (0.0%)	1 (100.0%)	1 (100.0%)
| Bone	4 (57.1%)	3 (42.9%)	7 (100.0%)
| Abdominal wall	1 (50.0%)	1 (50.0%)	2 (100.0%)
| Prostate	1 (100.0%)	0 (0.0%)	1 (100.0%)
| Lung/Pleura	0 (0.0%)	1 (100.0%)	1 (100.0%)

IQR: interquartile range.

**Table 4:** Detailed description of therapy classes in relation to primary NET.

Primary NET Site	Therapy						Total
	Chemotherapy		Everolimus	SSA	Tem/Cap, SSA, Xermelo^®^	Watch and Wait	
	Tem./Cap.	STZ/5FU					
Pancreas	6 (30.00%)	3 (15.00%)	4 (20.00%)	3 (15.00%)	0 (0%)	4 (20.00%)	20 (25.64%)
Intestine	0 (0%)	0 (0%)	2 (4.65%)	13 (30.23%)	0 (0%)	28 (65.12%)	43 (55.13%)
Prostate	0 (0%)	0 (0%)	0 (0%)	0 (0%)	0 (0%)	1 (100.00%)	1 (1.28%)
Lung	0 (0%)	0 (0%)	0 (0%)	1 (16.67%)	5 (83.33%)	0 (0%)	6 (7.69%)
Unclear	0 (0%)	0 (0%)	0 (0%)	7 (87.5%)	0 (0%)	1 (12.5%)	8 (10.26%)
Total	6 (7.69%)	3 (3.85%)	6 (7.69%)	24 (30.77%)	5 (6.41%)	34 (43.59%)	78 (100.00%)

NET: neuroendocrine tumor, Tem./Cap.: temozolomide and capecitabine (chemotherapy), SSA: somatostatin analogue, STZ/5FU: streptozocin and 5-fluorouracil (chemotherapy).

**Table 5:** Characteristics of therapy (any vs. no therapy) in relation to iodine content, to localization of primary tumor, and to primary tumor grade.

		Therapy		Total
		Any	None (Watch and Wait)	
Lesion NIC	Min/Max	5.1/77.2	7.7/97.5	5.1/97.5
	Med [IQR]	12.2 [9.4; 19.3]	16.9 [11.7; 27.6]	14.5 [10.0; 20.9]
	n	44	34	78
Hotspot NIC	Min/Max	6.8 / 100.6	10.9/141.8	6.8/141.8
	Med [IQR]	18.0 [14.3; 29.2]	27.1 [20.3; 38.9]	21.8 [15.9; 33.8]
	n	44	34	78
Primary NET Site	Pancreas	16 (80.00%)	4 (20.00%)	20 (25.64%)
	Intestine	15 (34.88%)	28 (65.12%)	43 (55.13%)
	Prostate	0 (0%)	1 (100.00%)	1 (1.28%)
	Lung	6 (100.00%)	0 (0%)	6 (7.69%)
	Unclear	7 (87.5%)	1 (12.5%)	8 (10.26%)
	n	44 (56.41%)	34 (43.59%)	78 (100.00%)
Primary NET Grade	G1	14 (43.75%)	18 (56.25%)	32 (41.03%)
	G2	20 (57.14%)	15 (42.86%)	35 (44.87%)
	G3	10 (90.91%)	1 (9.09%)	11 (14.10%)
	n	44 (56.41%)	34 (43.59%)	78 (100.00%)

NIC: normalized iodine content, min: minimum, max: maximum, med: median, IQR: interquartile range, SD: standard deviation, n: number of lesions.

## Data Availability

The data from this study can be presented upon request. Please contact the corresponding author.

## Supplementary Materials

The following supporting information can be downloaded at: https://www.mdpi.com/article/10.3390/curroncol30020115/s1, Figure S1. Distribution of normalized iodine content in each lesion across the total patient population (*n* = 26).
