# Supplementary figures and images for: Spectral Computed Tomography-Derived Iodine Content and Tumor Response in the Follow-Up of Neuroendocrine Tumors—A Single-Center Experience
Source: Curr Oncol. 2023 Jan 23;30(2):1502–15. doi: 10.3390/curroncol30020115 (PMC9954990; doi:10.3390/curroncol30020115)

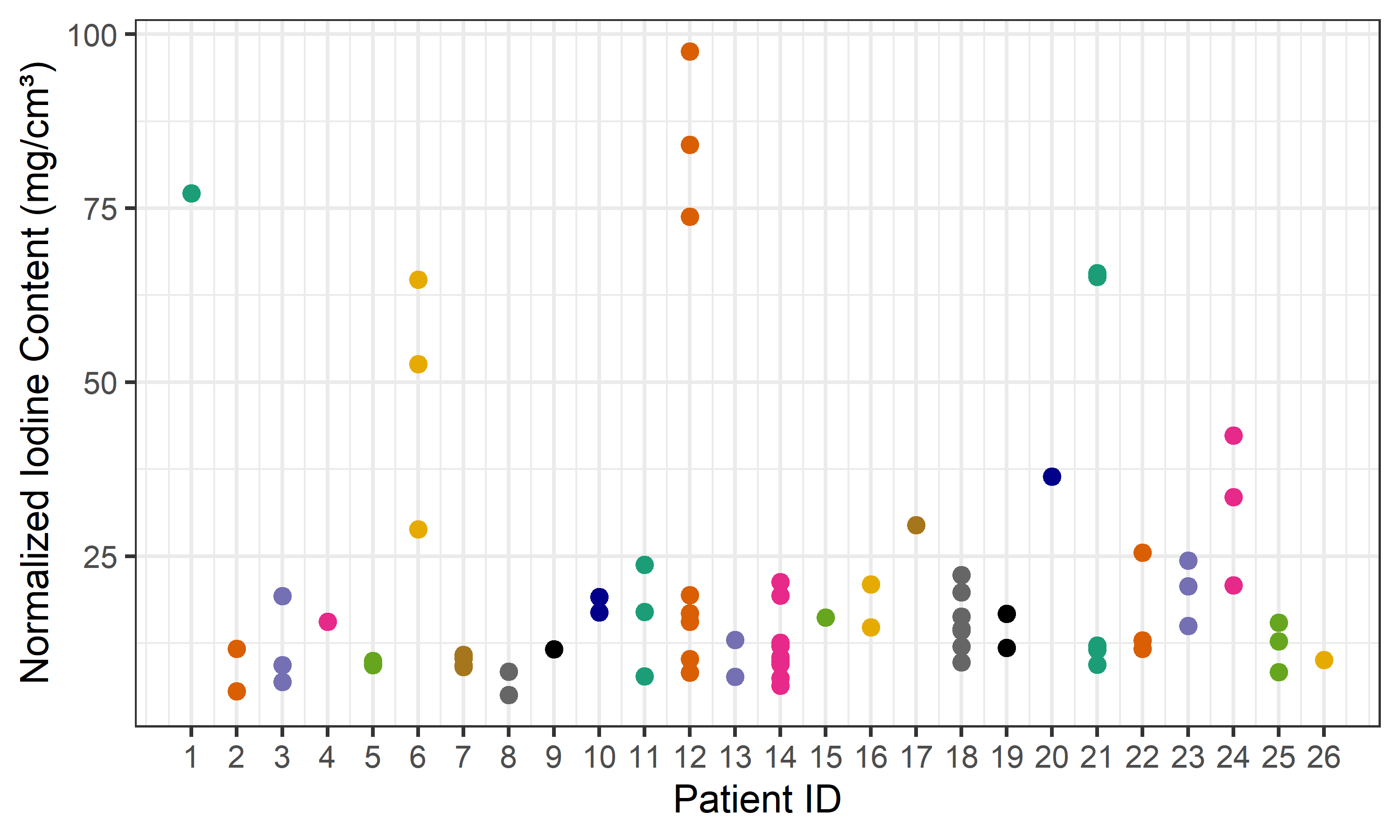

Supplement: Supplementary file 1 [file curroncol-30-00115-s001.zip › curroncol-2087498-supplementary-Figure S1.tiff]
